# Supplementary material for: Elasticity-controlled jamming criticality in soft composite solids
Source: Nat Commun. 2024 Feb 24;15:1691. doi: 10.1038/s41467-024-45964-y (PMC10894283; doi:10.1038/s41467-024-45964-y)
Supplement: Supplementary file 1 — Supplementary Information [file 41467_2024_45964_MOESM1_ESM.pdf]

Supplemental Information for
"Elasticity-Controlled Jamming Criticality in Soft Composite Solids"

Yiqiu Zhao,[1,*] Haitao Hu,[1] Yulu Huang,[1] Hanqing Liu,[2] Caishan Yan,[1] Chang Xu,[1] Rui Zhang,[1] Yifan Wang,[3] and Qin Xu[1,†]

[1]*Department of Physics, The Hong Kong University of Science and Technology, Hong Kong SAR, China*
[2]*Theoretical Division, Los Alamos National Laboratory, Los Alamos, New Mexico 87545, USA*
[3]*School of Mechanical and Aerospace Engineering, Nanyang Technological University, 639798, Singapore*

(Dated: January 15, 2024)

CONTENTS

I. Supplementary Methods 1
A. Microsphere inclusions 1
1. Material properties 1
2. Shear-jamming density ϕ_m of dense suspensions 1
3. Jamming strain for suspensions with $\phi > \phi_m$ 3
4. Frictionless jamming density ϕ_0 4
B. PDMS matrix 5
C. Multi-Axial Shear Test 6
1. Measuring protocol 6
2. Volume conservation 7

II. Supplementary References 10

I. SUPPLEMENTARY METHODS

A. Microsphere inclusions

1. *Material properties*

The polystyrene (PS) particles (XMO-50, Dongguan Xinmiao New Material Co.) have a size distribution consistent with a log-normal distribution $f(r) = \frac{1}{\sqrt{2\pi}\sigma r}\exp(-\frac{1}{2}(\frac{\ln(r/r_0)}{\sigma})^2)$, where $r_0 = 12$ μm and $\sigma = 0.6$ (Supplementary Fig. 1(a)). The mean radius of the PS particles is $\langle r\rangle = \exp(\ln r_0 + \sigma^2/2) = 14.4$ μm. We measured the distribution by counting 17,825 particles imaged with an optical microscope. The mass density of the PS particles was measured as $\rho = 1.047 \pm 0.002$ g/ml using the sedimentations method. Additionally, the size distribution of the glass spheres (G4649, Sigma-Aldrich Inc.) also coincides with the log-normal distribution with $r_0 = 20$ μm and $\sigma = 0.5$ (Supplementary Fig. 1(a)), resulting in a mean radius of $\langle r\rangle = 22.7$ μm. The mass density of the glass particles was 2.33 g/mL.

The particle shear modulus (G_p) was measured using a high-precision nanoindenter (Hysitron TI-980, Bruker). The particles were compressed between a flat-ended diamond probe (TI-0145, Bruker) and a flat glass substrate. The representative plots of the indentation force (F) against the indentation displacement (δ) are shown in Supplementary Fig. 1(b). For measuring the PS particles, the deformations of both the diamond probe and the glass substrate were negligible. We fitted the indentation curves using the following Hertzian model

$$F = \frac{4}{3}E_p^* R^{\frac{1}{2}}(\frac{\delta}{2})^{\frac{3}{2}}, \quad (1)$$

where R is the particle radius and E_p^* is the only fitting parameter. From independent measurements on 5 particles with radii ranging from 10 to 15 μm, we obtained $E_p^* = 4.9 \pm 0.6$ GPa. Using the relationships $E_p^* = E_p/(1-\nu_p^2)$, where E_p and ν_p are the Young modulus and the Poisson ratio of the particles, respectively, and $G_p = E_p/2(1+\nu_p)$, we have

$$G_p = \frac{1-\nu}{2}E_p^*. \quad (2)$$

Given that the Poisson ratio of PS was around 0.35 at room temperature [1, 2], we obtained $G_p = 1.6 \pm 0.2$ GPa for the PS particles.

For measuring the glass particles, we assumed that the glass substrate had the same elastic modulus as the particles and neglected the deformations of the diamond probe. Therefore, the Hertzian contact model becomes

$$F = \frac{4}{3}E_p^* R^{\frac{1}{2}}(\frac{\delta}{1+2^{\frac{2}{3}}})^{\frac{3}{2}}. \quad (3)$$

By averaging the results of 12 particles with radii ranging from 5 to 20 μm, we obtained $E_p^* = 40 \pm 13$ GPa. Considering that the Poisson ratio of the Sigma-Aldrich glass beads is 0.21, we obtained $G_p = 15.8 \pm 5.1$ GPa.

2. *Shear-jamming density ϕ_m of dense suspensions*

PS-PDMS suspensions — The steady-state rheology of the PS-PDMS suspensions was measured using a rheometer (MCR 302, Anton Paar) equipped with a parallel plate geometry. Both the top and bottom plates are made

* yiqiuzhao@ust.hk
† qinxu@ust.hk

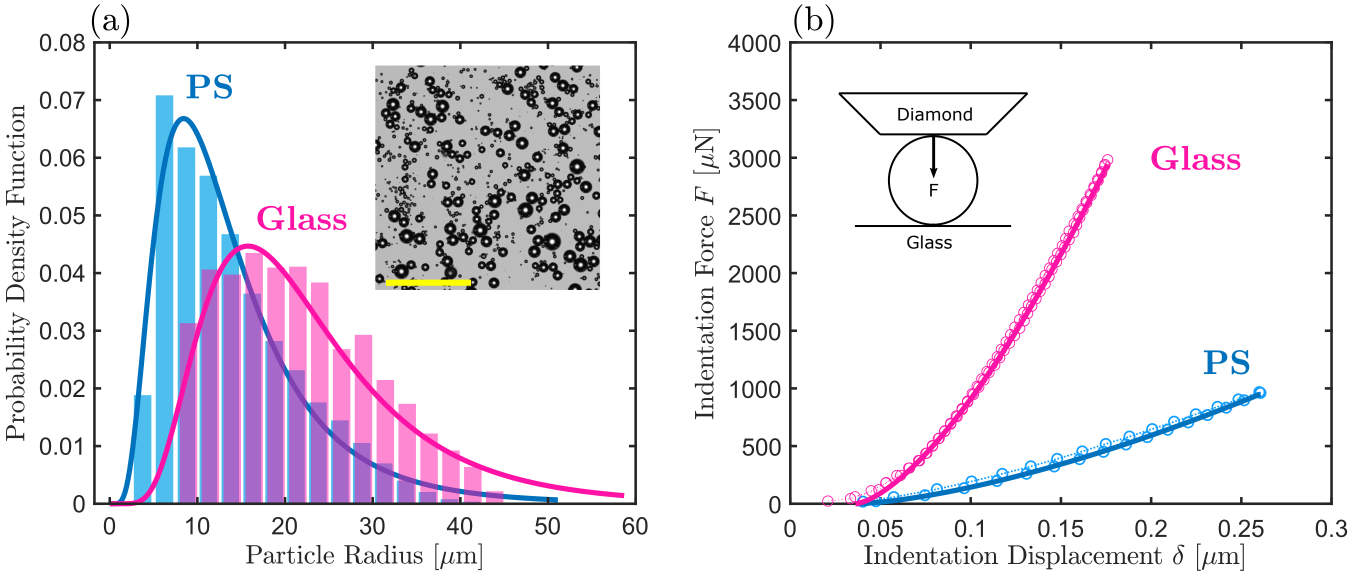

Supplementary Fig. 1. Size distribution and shear modulus of the polystyrene (PS) and glass microspheres. (a) Probability distribution of the particle radius for the PS and glass microsphere particles. The solid blue and magenta curves are log-normal functions $f(r) = \frac{1}{\sqrt{2\pi}\sigma r}\exp(-\frac{1}{2}(\frac{\ln(r/r_0)}{\sigma})^2)$, where for the PS particles $r_0 = 12$ μm and $\sigma = 0.6$, and for the glass particles $r_0 = 20$ μm and $\sigma = 0.5$. The inset panel shows a microscope image of a layer of dry PS powder in a Petri dish, demonstrating their spherical shape. The yellow scale bar represents 500 μm. (b) Two representative force–displacement curves measured from nano-indentation experiments for a glass particle and a PS particle. Each curve represents data measured during a compression–decompression cycle. The magenta and light blue curves are the best fits using Supplementary Eq. 1 and Supplementary Eq. 3, respectively. An offset of δ was used in the fit due to the uncertainty of the zero-point. The experimental setup is sketched in the inset.

of flat glasses. The top shear plate (PP43/GL, Anton Paar) has a radius of 21.5 mm, and the gap distance was fixed to 1.5 mm. Prior to rheological measurements, the suspensions were pre-sheared at a fixed shear stress $\tau = 80$ Pa for 1 hour, followed by a gradual decrease of shear stress until $\tau = 0.1$ Pa. Next, the flow curves were measured in the stress-controlled approach by decreasing τ from 80 Pa to 0.16 Pa. Figure 2(a) shows the flow curves with varying particle volume fractions (ϕ), with the plot of $\phi = 0$ representing the Newtonian flow curve of the base PDMS. Above $\tau = 80$ Pa, the capillary pressure at the liquid-air interface was not enough to confine the suspensions. The dashed lines indicate a plateau relative viscosity ($\eta_{\mathrm{plateau}}/\eta_{\mathrm{s}}$) for different ϕ. Theses values of $\eta_{\mathrm{plateau}}/\eta_{\mathrm{s}}$ were reported in both the main text and Supplementary Fig. 2(d). The results of $\eta_{\mathrm{plateau}}(\phi)/\eta_{\mathrm{s}}$ in Supplementary Fig. 2(d) were fitted to the Krieger–Dougherty relation with $\phi_{\mathrm{J}} = 0.594 \pm 0.003$, consistent with ϕ_{m} in Eq. 7 of the main text.

We interpret the shear thinning observed for $\phi = 0.53$ and 0.55 below $\tau = 1$ Pa as a transient rheological response of dense PS-PDMS suspensions. To demonstrate this, Supplementary Fig. 3 presents the flow curves of PS-PDMS suspensions having $\phi = 0.55$ characterized under different measuring rates. By defining t_{w} as the measuring duration at each τ, we observed a gradual transition of flow curves towards Newtonian-like behaviors as t_{w} increased from 100 s to 2 hours. Since the uncrosslinked PDMS solvents are made of longer polymer chains, their relaxation within dense suspensions can be very slow, particularly under low shear stresses. If the measuring period was extended significantly, we expected that the relative viscosity η/η_{s} below $\tau = 1$ Pa would align with that within the plateau regime, 1 Pa $< \tau <$ 10 Pa. Thus, the plateau viscosities $\eta_{\mathrm{plateau}}(\phi)$ were used to determine the shear-jamming volume fraction ϕ_{J} in Supplementary Fig. 2(d).

PS-water/glycerol suspensions — We also characterized the steady-state rheology of PS particles dispersing in a density-matched aqueous glycerol [3]. The solvent viscosity is $\eta_{\mathrm{s}} = 1.75$ mPa·s. The samples were sufficiently pre-sheared before each measurement. Figure 2(b) shows the flow curves for different ϕ. Each curve was obtained by averaging three independent measurements. Figure 2(e) shows the maximum relative viscosity $\eta_{\mathrm{max}}/\eta_{\mathrm{s}}$ as a function of ϕ. The data were fitted to the Krieger–Dougherty relation with $\phi_{\mathrm{J}} = 0.599 \pm 0.001$. The PS-water/glycerol suspensions exhibited classical shear thickening behaviors. Considering the shear thickening mechanism in the suspension rheology [4], we interpret $\phi_{\mathrm{J}} = 0.599$ as the minimum particle volume fraction needed to observe frictional shear jamming in PS-water/glycerol suspensions. As this ϕ_{J} is consistent with that obtained from PS-PDMS suspensions, we expect that the shear-jamming of the PS particles in PDMS is also governed by frictional

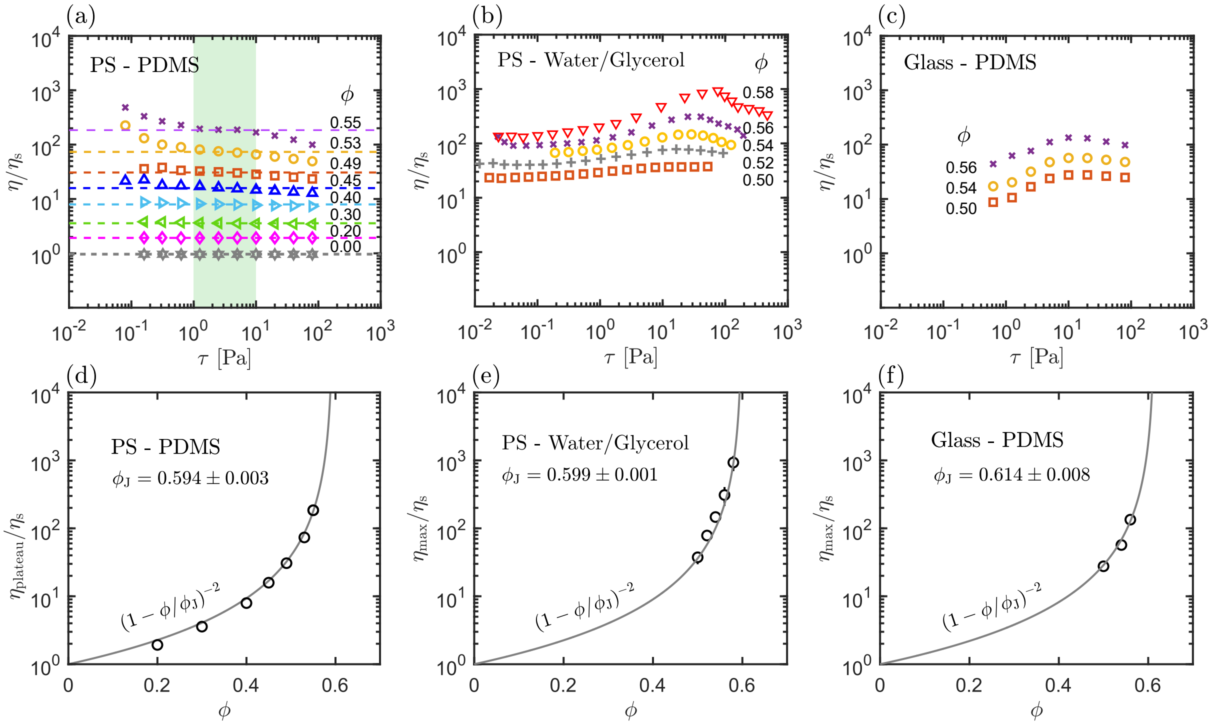

Supplementary Fig. 2. Steady-state rheology to measure the frictional jamming point of dense suspensions. (a–c) The flow curves of steadily sheared PS particles dispersing in the PDMS silicone base fluid (a), in the density-matched 20 wt% glycerol aqueous solution (b), and of glass beads dispersing in the PDMS silicone base fluid (c). In panel (a), the regime between 1 Pa and 10 Pa, where the flow curves display a plateau, is highlighted in light green. The dashed horizontal lines for each volume fraction represent the plateau relative viscosities. (d–f) The plateau relative viscosities (d) and the maximum relative viscosities (e–f) of the flow curves shown in (a–c), respectively, are presented. The solid gray curves in (d–f) represent the best fits using the Krieger–Dougherty relation, $(1-\phi/\phi_J)^{-2}$, with the fitted ϕ_J shown in the legend.

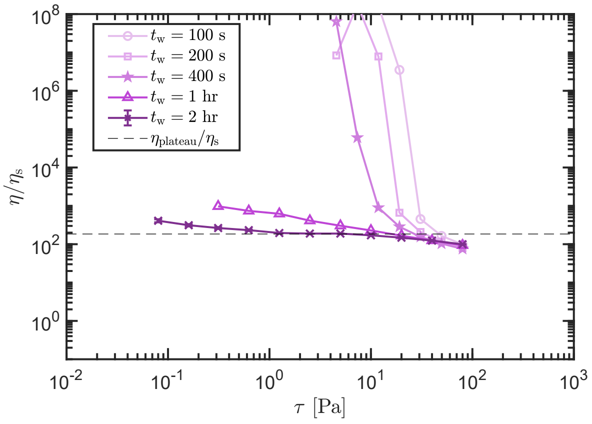

Supplementary Fig. 3. Flow curves of the PS-PDMS suspension (ϕ = 0.55) characterized with different measuring rates. For each shear stress τ, the relative viscosity η/η_s was determined by averaging the measured viscosity over a period of t_w. The waiting time t_w was varied systematically from 100 s to 2 hours.

contacts.

Glass-PDMS suspensions — The rheology of glass-PDMS suspensions was measured using the same protocol as that for PS-PDMS suspensions. The minimum applied stress was $\tau = 0.625$ Pa, which corresponds to the sedimentation stress of a glass bead in a PDMS solvent. The flow curves of glass-PDMS suspensions are shown in Supplementary Fig. 2(c). Figure 2(f) shows the maximum relative viscosity η_{max}/η_s as a function of ϕ. The data were fitted to the Krieger–Dougherty relation with $\phi_J = 0.614 \pm 0.008$.

3. *Jamming strain for suspensions with $\phi > \phi_m$*

Figure 4(a) illustrates the protocol of measuring the critical jamming strain (ε). Initially, the gap between the parallel plates was filled with a suspension ($\phi > \phi_m$), and the gap size was set to $d_0 = 1.5$ mm (state 1). The top plate was then lifted to a new position with $d_1 > d_0$ (state 2). To fully relax the suspension, an oscillatory shear was applied with an angular frequency $\omega = 10$ rad/s and a strain amplitude $\delta\gamma_a = 10\%$. The resulting state (state 3) was considered as the reference state ($\varepsilon = 0$). Next, the top plate was lowered back to the initial position $d_0 = 1.5$ mm (state 4). The axial strain ε with respect to the reference state was calculated as $(d_1 - d_0)/d_1$. In this

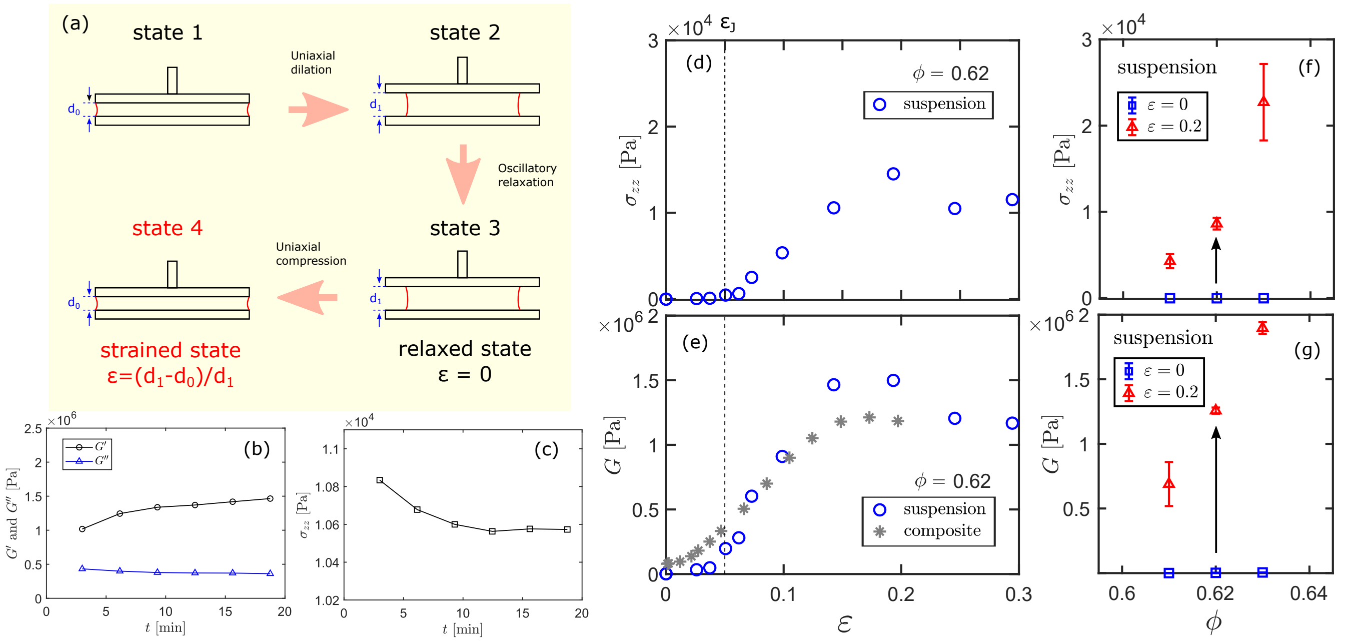

Supplementary Fig. 4. **Strain-induced rigidity transition in PS-PDMS suspensions.** (a) Schematic of the experimental protocol to measure shear-jamming transitions in PS-PDMS suspensions. The parallel-plate was initially filled up with a suspension under a gap size $d_0 = 1.5$ mm (state 1). The top plate was then lifted up to a new position with $d_1 > d_0$ (state 2). At this position, the suspension was fully relaxed due to an oscillatory shear with an angular frequency $\omega = 10$ rad/s and a strain amplitude $\delta\gamma_a = 10\%$ for 30 mins (state 3). State 3 was considered as the reference state ($\varepsilon = 0$). Finally, the top plate was lowered again to the initial position with a gap size d_0 (state 4), and the resulting axial strain is $\varepsilon = (d_1 - d_0)/d_1$. (b) Time evolution of the storage modulus (G') and loss modulus (G'') measured in state 4 with $d_1 = 1.76$ mm. An angular frequency of $\omega = 0.1$ rad/s and a strain amplitude of $\delta\gamma_a = 0.01\%$ were applied during the oscillatory tests. The plateau values of G' are denoted as the shear modulus of the jammed suspensions (G). (c) Time evolution of the normal stress (σ_{zz}) measured in state 4. (d – e) Normal stress σ_{zz} and shear modulus G of a suspension with $\phi = 0.62$ as the function of ε (blue circles). Both σ_{zz} and G begin to become nonzero near $\varepsilon_J = 0.05$, indicating a shear jamming transition. The gray stars in (e) show the strain-dependent shear modulus of a PS-PDMS composite sample with $\phi = 0.62$ and $G_m = 1.28$ kPa. (f – g) Normal stress σ_{zz} and shear modulus G of suspensions measured under $\varepsilon = 0$ and $\varepsilon = 0.2$, respectively, for samples with $\phi > \phi_J$. The black arrow shows the shear jamming process for $\phi = 0.62$ induced by an axial strain.

compressed state (state 4), we measured the shear modulus of the jammed suspension through an oscillatory shear with $\omega = 0.1$ rad/s and $\delta\gamma_a = 0.01$ %. For jammed suspensions, the storage modulus (G') remained larger than the loss modulus (G'') (Supplementary Fig. 4(b)), and a positive normal stress (σ_{zz}) appeared simultaneously (Supplementary Fig. 4(c)). For instance, we measured the critical jamming strain for a PS-PDMS suspension with $\phi = 0.62$. As shown in Figs. 4(d) and (e), both σ_{zz} and G begin to become non-zero at $\varepsilon = 0.05$, which is consistent with the prediction from Eq. 7 in the main text. This critical strain ($\varepsilon = 0.05$) indicates the onset of a rigidity transition in the PS-PDMS suspension. Figure 4(e) also shows the shear modulus ($G(\varepsilon)$) of a cured PS-PDMS composite with $\phi = 0.62$ and $G_m = 1.28$ kPa (grey stars), closely matching that of an uncured PS-PDMS suspension with the same particle volume fraction (blue circles).

To further demonstrate that the PS-PDMS suspensions were unjammed at $\varepsilon = 0$, Figs. 4(f) and (g) show that the normal stresses (σ_{zz}) and shear moduli (G) of the suspensions with $\phi = 0.61$, 0.62, and 0.63($> \phi_J$) rise from zero to finite values, respectively, as ε increases from 0 to 0.2. Additionally, Supplementary Fig. 5 demonstrates that the suspension with $\phi = 0.61 > \phi_J$ can undergo a jamming transition under a simple shear with constant shear stresses. The regimes with non-zero shear rates ($\dot{\gamma}$) in Supplementary Fig. 5 suggest that the PS-PDMS suspensions with $\phi = 0.61$ were initially unjammed.

4. *Frictionless jamming density ϕ_0*

The frictionless jamming densities (ϕ_0) for both polydisperse PS and glass particles differ noticeably from the value of 0.64 for monodisperse spheres [5]. To investigate this difference, we conducted molecular dynamics (MD) simulations for frictionless Hertzian spheres using the Large-scale Atomic/Molecular Massively Parallel Simulator (LAMMPS) program [6]. The particle interaction

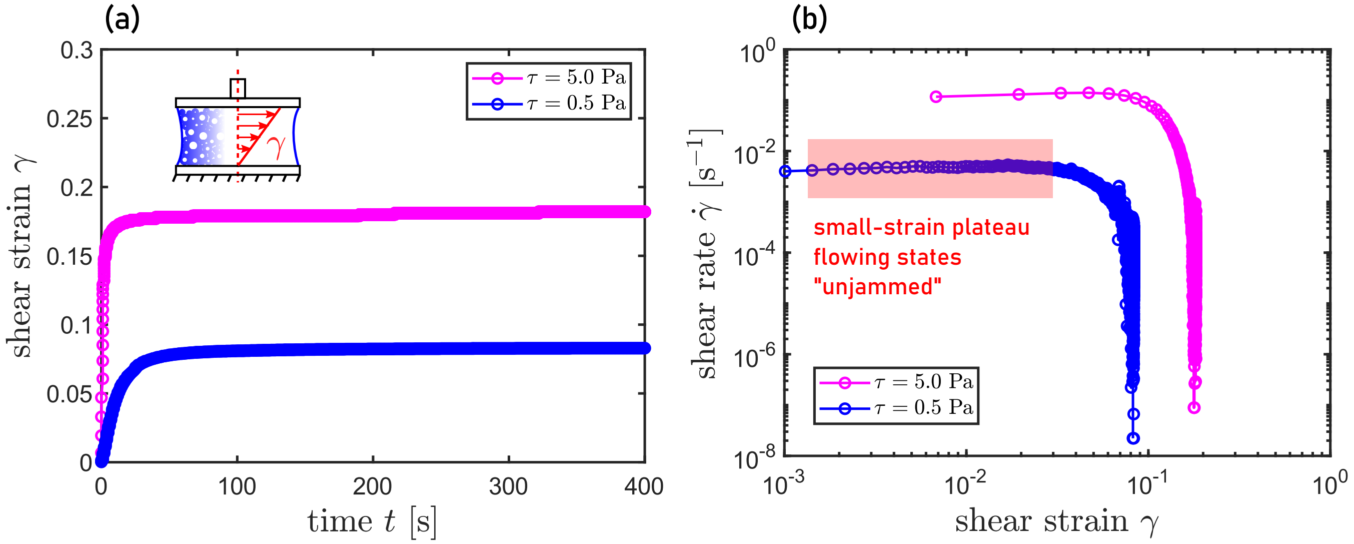

Supplementary Fig. 5. Liquid-to-solid transition of PS-PDMS suspensions ($\phi = 0.61$) induced by shear. (a) Plots of the accumulative shear strain γ against time t under constant shear stresses, $\tau = 0.5$ Pa and 5.0 Pa, respectively. (b) Plots of shear rate $\dot{\gamma}$ against shear strain γ under the two different shear stresses.

was governed by the frictionless Hertzian contact law

$$\mathbf{F}_{ij} = \sqrt{\delta}\sqrt{\frac{R_i R_j}{R_i + R_j}}(k_\mathrm{n}\delta\mathbf{n}_{ij} - m_\mathrm{eff}\gamma_\mathrm{n}\mathbf{v}_\mathrm{n}), \qquad (4)$$

where $\mathbf{F}_{ij}$ is the force exerted on the ith particle by the jth particle, R_i and R_j represent the radii of the two particles, δ is the overlap distance, $k_\mathrm{n} = 3.3 \times 10^3$ N m^{-1} is a spring constant, $\gamma_\mathrm{n} = 10^{-5}$ s^{-1} is a damping constant, $m_\mathrm{eff} = m_i m_j/(m_i + m_j)$ is the effective mass of two particles having masses of m_i and m_j, and $\mathbf{n}_{ij}$ is the unit vector along the line connecting the centers of the two particles. The mass of the particles in our simulations was set to 6×10^{-13} kg. A global damping force $\mathbf{F}_\mathrm{d} = -\gamma_\mathrm{d}\mathbf{v}$ with $\gamma_\mathrm{d} = 10^{-8}$ kg s^{-1} was exerted on each particle to dissipate the kinetic energy of the system.

We simulated the frictionless jamming transition using a quasi-static isotropic compression protocol. During each simulation, an initial packing of 2048 particles was randomly generated within a cubic box (Supplementary Fig. 6(a)). The particles have the same size distribution as that was used in the experiments (Supplementary Fig. 6(b)). The box size was chosen such that $\phi = 0.616$ for the PS particles and 0.610 for the glass particles initially, where periodic boundary conditions were used in all three directions.

After the initial generation, the systems were relaxed by particle interactions and global damping for $t = 50\,\mu$s with a small time step of $dt = 0.0001\,\mu$s. Ten compression simulations were performed for both the PS and glass systems, with identical particle size distributions but different initial configurations. The simulation box was compressed in all three directions with a strain rate of $\dot{\epsilon} = 6.25 \times 10^{-2}$ s^{-1}, varying the box length in accordance with the equation $L(t) = L_0(1 - \dot{\epsilon}t_e)$, where $t_e = 8 \times 10^{-3}$s. After each compression step, the system was allowed to fully relax.

The equilibrium pressure and non-rattler contact number were measured for each step, and both showed a transition at ϕ_0 (Figs. 6(c) and (d)). The non-rattler coordination number is defined as $C^* = M_4/N_4$, where M_4 is the total contact number of the N_4 particles having at least four contacts. This coordination number jumps to the isostatic value of 6 at ϕ_0, as expected for the emergence of rigidity. The result shows that the system reached a jamming state for the PS particles at a volume fraction of 0.6896 ± 0.0006 and for the glass beads at a volume fraction of 0.6766 ± 0.0006. The uncertainty here corresponds to the incremental step size of the volume fraction.

B. PDMS matrix

To determine the shear modulus of PDMS matrix (G_m), we measured the storage modulus using oscillatory shear with an angular frequency $\omega = 0.1$ rad/s and a strain amplitude $\delta\gamma_a = 1\%$. Figure 7 shows the plot of G_m against k, and we found that

$$G_\mathrm{m} = A(k - k_\mathrm{c})^a, \qquad (5)$$

where $A = 2.396$ GPa, $k_\mathrm{c} = 0.0055$, and $a = 2.572$. To fabricate a soft composite with a specific G_m, we prepared the density of crosslinkers based on Supplementary Eq. 5. Figures 8 shows a representative cross-section image of a PS-PDMS composite with $G_\mathrm{m} = 1.28$ kPa and $\phi = 0.57$. Each sample has a cylindrical shape with an initial radius $R_\mathrm{s} = 10$ mm and a height $H = 10$ mm, and contains approximately $\sim 10^8$ particles.

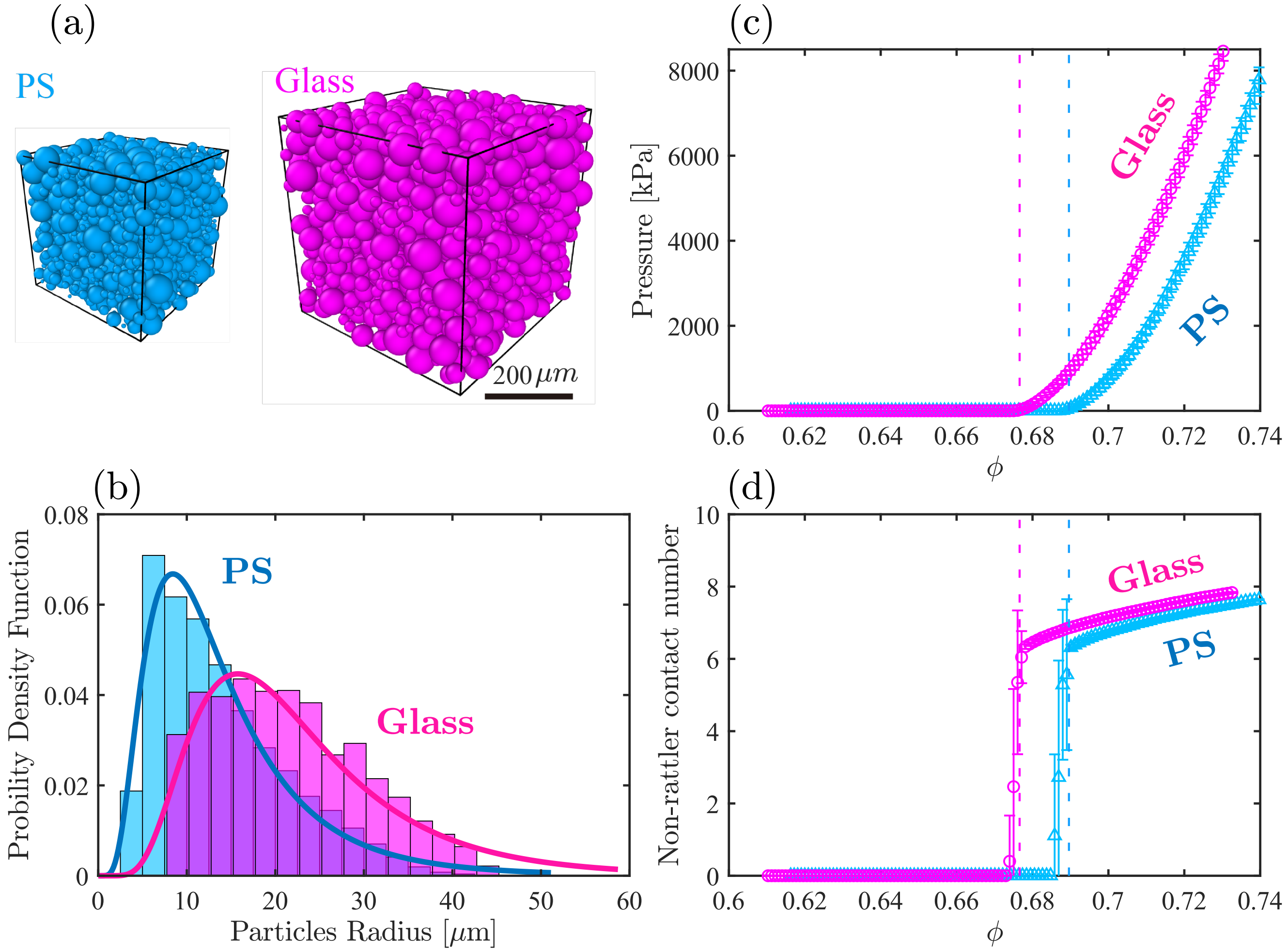

Supplementary Fig. 6. **Numerical simulation to measure the random close packing ϕ_0 for the PS and glass microspheres.** (a) The polystyrene (PS) and glass granular systems were simulated using repulsive Hertzian spheres having the same size distributions as that used in experiments. Initially, the volume fraction was $\phi = 0.616$ for the model PS particles and is 0.610 for the model glass particles. Each model system contains 2,048 particles. (b) The size distributions of the spheres used in the simulations were designed to match that used in the experiments. The two solid curves are copied from Supplementary Fig. 1(a). (c) Pressure and (d) non-rattler contact number as functions of the volume fraction for both the model PS and glass granular systems. The error bars indicate the standard deviations from 10 simulations with different particle configurations. The two vertical dashed lines indicate $\phi_0 = 0.6896$ and 0.6766 for the PS and glass spheres, respectively.

C. Multi-Axial Shear Test

1. *Measuring protocol*

The relationship between G and ε was characterized through a multi-axial shear test. When d decreased, no torsional stress was applied. For most experiments, the step size δd was set to 0.2 mm for $\varepsilon < 0.25$ and to 0.5 mm for larger strains. At a fixed gap size d, the top plate applied an oscillatory shear to the samples. The shear modulus of composites (G) showed a transient relaxation until it reached a plateau (Supplementary Fig. 9). We reported the shear moduli measured 20 minutes after each compression when the system has reached the equilibrium.

During the oscillatory tests, the angular position of the shear plate was set to follow $\theta(t) = (\delta\gamma_a d/R_p)\sin\omega t$ with $\delta\gamma_a = 0.01$ %. The apparent shear strain on the sample was $\delta\gamma = (R_s/R_p)\delta\gamma_a$, which slightly increased from 0.0047% to 0.0053% as d decreased from 10 mm to 8 mm due to a small increase in R_s. Figure 10 shows the results of the PS-PDMS with $G_m = 0.35$ kPa and $\phi = 0.57$ under different axial strains. The dashed lines indicate $\delta\gamma$ and ω at different gap sizes. Considering the difference between the radius of the shear plate (R_p) and that of the samples (R_s), we calculated the storage modulus from the rheometer output $G' = (R_p/R_s)^4 G'_{\rm rheo}$. The shear moduli of dense composites were defined by G' measured at $\omega = 0.1$ rad/s. Figure 11 shows that $\omega = 0.1$ rad/s is sufficiently small to capture the low-frequency plateau of G'. The third row in Supplementary Fig. 10 shows the

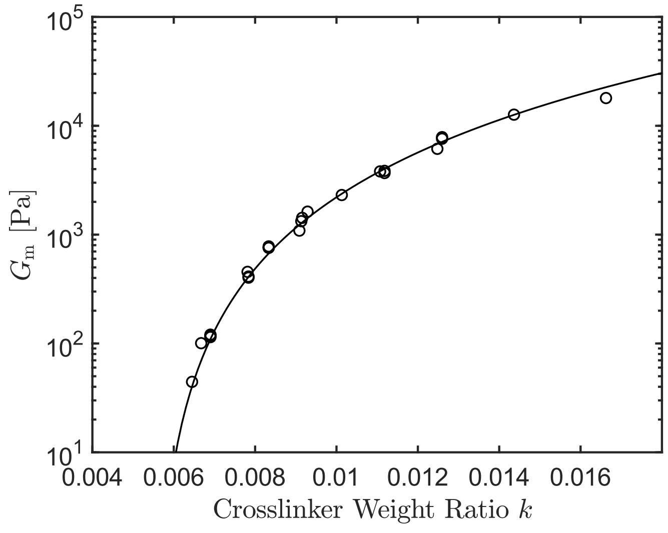

Supplementary Fig. 7. **Matrix shear modulus controlled by the crosslinking density.** The shear moduli of the PDMS gels (G) with different crosslinking densities (k) are presented. The dashed black curve is the best fit to the empirical power law $G_m = A(k - k_c)^a$ with $A = 2.396$ GPa, $k_c = 0.0055$, and $a = 2.572$.

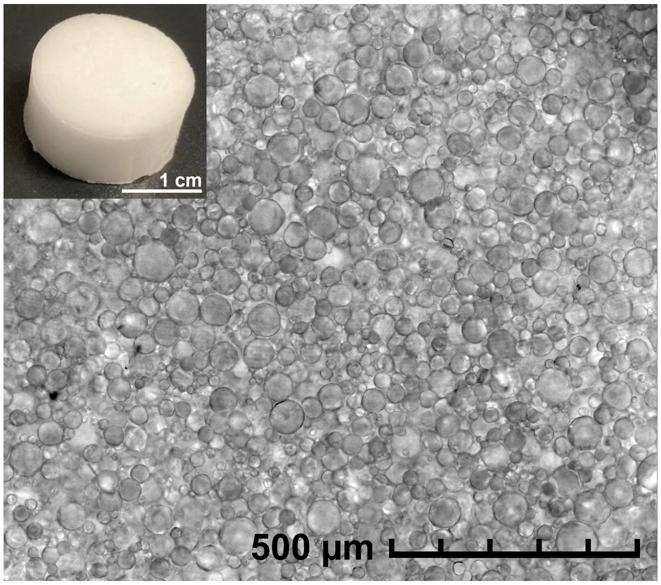

Supplementary Fig. 8. **Representative PS-PDMS composite.** Inset panel: A PS-PDMS composite sample with $\phi = 0.57$ and $G_m = 1.28$ kPa. Main panel: A microscope image of the cross section of the sample shown in the inset panel.

overlay of 10 stress–strain cycles of a PS-PDMS composite with $G_m = 0.35$ kPa and $\phi = 0.57$ at different gap sizes. All the curves are stable ellipses, consistent with the expectation for a viscoelastic material. Therefore, no evidence of wall slip was found. Due to the strong adhesion between the samples and the shear plate, wall slip was even avoided without normal stress.

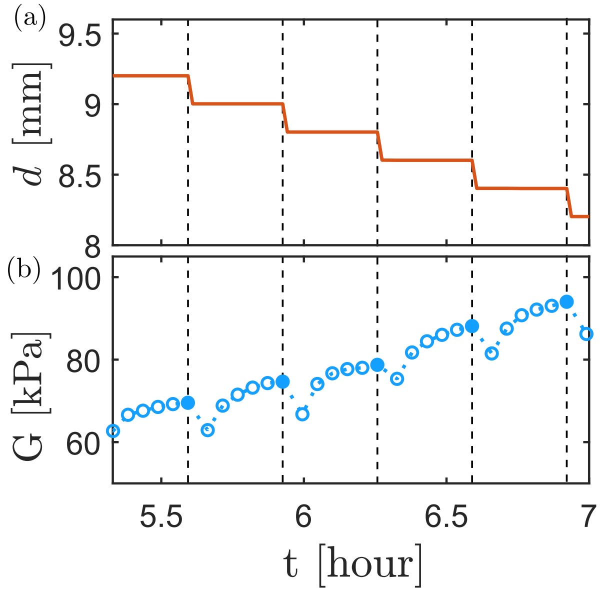

Supplementary Fig. 9. **Transient relaxations after each compression step.** Plots of the temporal evolution of the gap size d (a) and the shear modulus G (b) for a soft composite with $\phi = 0.57$ and $G_m = 1.28$ kPa. We reported the equilibrium values measured 20 min after each compression. The equilibrium values are indicated by the filled circles in (b).

2. *Volume conservation*

The volume of the composite remained unchanged under vertical compressions. To confirm this statement, a Thorlab camera was used to image the samples from the side. Figures 12(a–d) show four snapshots of a PS-PDMS composite ($\phi = 0.60$ and $G_m = 1.28$ kPa) under different axial strains. The boundaries detected using image analysis (yellow curves) show that the sample diameter varies with the gap size. The points in Supplementary Fig. 12(e) show the mean diameter D as a function of the sample height H for different ϕ and a constant $G_m = 1.28$ kPa. If the volume of a cylinder (V) is conserved under compression,

$$D = 2\sqrt{\frac{V}{\pi H}}. \tag{6}$$

The black curve in Supplementary Fig. 12(e) plots Supplementary Eq. 6 with $V = 3142$ mm^3, which is the initial volume of the sample. Since all the data points nicely fall on the black curve in Supplementary Fig. 12(e), the volume of composites must be conserved under compressions. Similarly, we have experimentally verified that the volume of an uncured PS-PDMS suspension remains conserved under axial strains (see *Supplementary Video 1*).

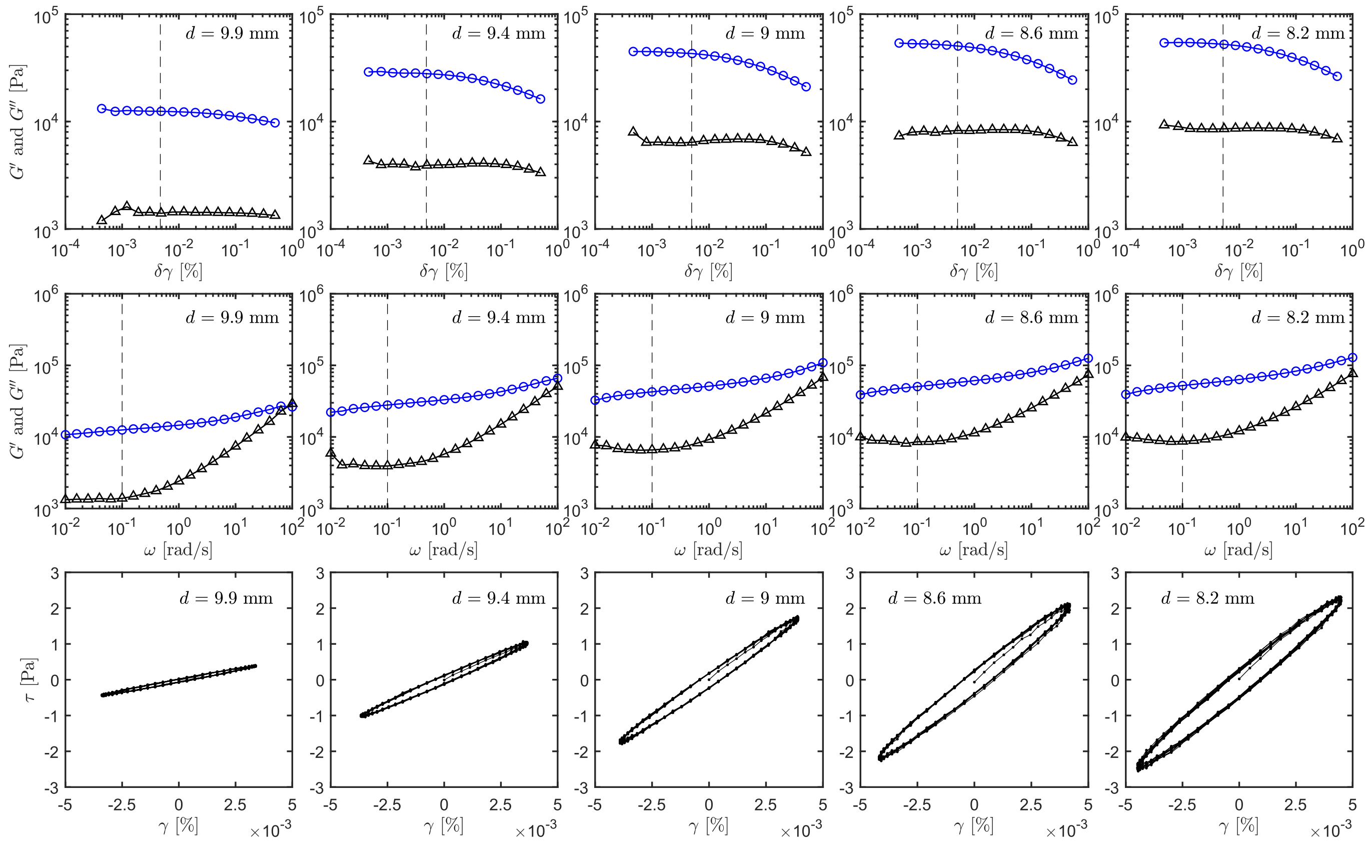

Supplementary Fig. 10. **Strain and frequency sweep for a composite sample under different axial strains.** The first row displays the storage modulus G' (blue circles) and the loss modulus G'' (black triangles) measured using an oscillatory shear with a fixed angular frequency of $\omega = 0.1$ rad/s and varying strain amplitudes for a PS-PDMS composite ($\phi = 0.57$ and $G_{\mathrm{m}} = 0.35$ kPa) under different gap sizes. The vertical dashed lines mark the strain amplitude ($\delta\gamma_a = 0.01\%$) used to determine the composite shear modulus. The second row shows G' (blue circles) and G'' (black triangles) measured for the same composite with different ω using a constant $\delta\gamma$ indicated by the vertical dashed lines in the first row. The dashed lines in the second row represent the angular frequency ($\omega = 0.1$ rad/s) used to determine the composite shear modulus. The third row displays the overlay of 10 stress-strain cycles using the $\delta\gamma$ and ω indicated by the dashed lines in the first two rows. All the stress-strain loops resemble typical viscoelastic responses and show no sign of wall slip.

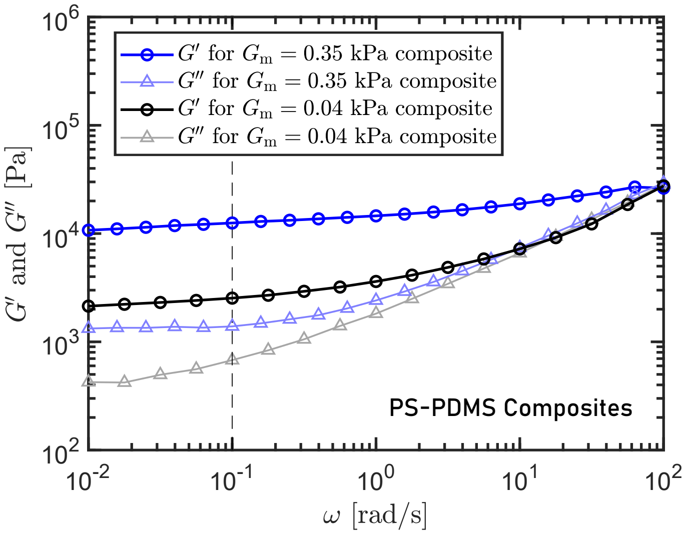

Supplementary Fig. 11. Oscillatory rheology of PS-PDMS composites (ϕ = 0.57) with different matrix stiffnesses (G_m = 0.35 kPa and 0.04 kPa).

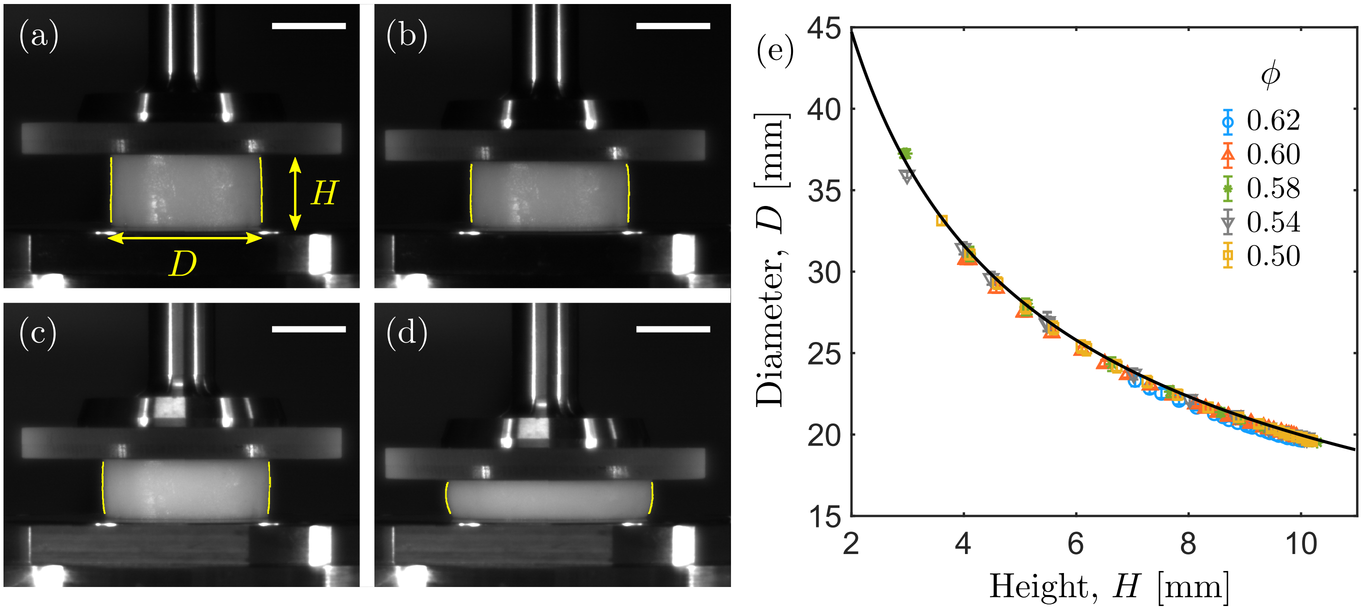

Supplementary Fig. 12. **Volume-conserving compressions.** (a–d) The snapshots of a PS-PDMS composite (ϕ = 0.60 and G_m = 1.28 kPa) subjected to different axial compressions, H = 9.6 mm (a), 8.7 mm (b), 7.9 mm (c), and 5.1 mm (d), respectively, are shown. The yellow lines are lateral boundaries detected by image analysis. Scale bars: 10 mm. (e) Plots of the sample diameter D against the sample height H for different ϕ. The error bars indicate the uncertainty in measuring D. The black line represents the prediction from Supplementary Eq. 6 with $V = 3142$ mm^3.

II. SUPPLEMENTARY REFERENCES

[1] Ryusuke Kono, "The dynamic bulk viscosity of polystyrene and polymethyl methacrylate," Journal of the Physical Society of Japan **15**, 718–725 (1960).

[2] P. H. Mott, J. R. Dorgan, and C. M. Roland, "The bulk modulus and Poisson's ratio of "incompressible" materials," Journal of Sound and Vibration **312**, 572–575 (2008).

[3] Koichi Takamura, Herbert Fischer, and Norman R. Morrow, "Physical properties of aqueous glycerol solutions," Journal of Petroleum Science and Engineering **98-99**, 50–60 (2012).

[4] Jeffrey F. Morris, "Shear thickening of concentrated suspensions: Recent developments and relation to other phenomena," Annual Review of Fluid Mechanics **52**, 121–144 (2020).

[5] Robert S. Farr and Robert D. Groot, "Close packing density of polydisperse hard spheres," The Journal of Chemical Physics **131**, 244104 (2009).

[6] Steve Plimpton, "Fast parallel algorithms for short-range molecular dynamics," Journal of Computational Physics **117**, 1–19 (1995).
