# Supplementary material for: Elasticity-controlled jamming criticality in soft composite solids
Source: Nat Commun. 2024 Feb 24;15:1691. doi: 10.1038/s41467-024-45964-y (PMC10894283; doi:10.1038/s41467-024-45964-y)
Supplement: Supplementary file 3 — Description of Additional Supplementary Files [file 41467_2024_45964_MOESM3_ESM.pdf]

<u>Description of Additional Supplementary Files</u>

Supplementary Movie Legend

Supplementary Movie 1: The video demonstrates that the total volume of a PS-PDMS suspension with a particle volume fraction of φ = 0.61 remains constant under axial strains. Initially, the sample was placed within a parallelplate cell with a gap size of d = 1.5 mm and then uniaxially stretched to d = 2 mm. After undergoing oscillatory relaxation at d = 2 mm, the sample was compressed back to d = 1.5 mm.
